# Comparative Analysis of Influenza Epidemiology Before and After the COVID‐19 Pandemic in Argentina (2018–2019 vs. 2022–2023)

**DOI:** 10.1111/irv.70078

**Published:** 2025-02-17

**Authors:** Angela Gentile, María del Valle Juárez, Gabriela Ensinck, Oscar Lopez, Pablo Melonari, Tatiana Fernández, Andrés Gioiosa, Gustavo Lazarte, Silvina Lobertti, María Florencia Lucion, Natalia Pejito, Camila Racana, Leandro López, Gabriela Gregorio

**Affiliations:** ^1^ Epidemiology Hospital de Niños “Ricardo Gutiérrez” Buenos Aires Argentina; ^2^ Infectious diseases Hospital de Niños “Dr. Víctor J. Vilela” Rosario Argentina; ^3^ Infectious diseases Hospital Pediátrico “Dr. Fernando Barreyro” Posadas Argentina; ^4^ Infectious diseases Hospital Pediátrico “Dr. Humberto Notti” Mendoza Argentina; ^5^ Infectious diseases Hospital Nacional “Profesor Alejandro Posadas” Buenos Aires Argentina

**Keywords:** ALRI, case fatality rate, influenza, influenza vaccine coverage

## Abstract

**Introduction:**

The COVID‐19 pandemic altered the epidemiology of respiratory viruses other than SARS‐CoV‐2. This study investigated the clinical‐epidemiological pattern of hospitalized pediatric patients with acute lower respiratory tract infections (ALRI) and influenza in Argentina, comparing prepandemic and postpandemic periods.

**Materials and Methods:**

This multicenter, cross‐sectional study included patients under 18 years old admitted for ALRIs in five tertiary centers of Argentina before (2018 and 2019) and after (2022 and 2023) COVID‐19. Changes in viral detection rates, seasonality, and case fatality rate (CFR), along with epidemiological and clinical characteristics, were analyzed. Indirect immunofluorescence assay (IFA) or RT‐PCR was used for virological diagnosis pre‐pandemic, and only RT‐PCR in post‐pandemic. Epi Info 7 and SPSS 15.0 was used for data analysis.

**Results:**

A total of 5838 cases of ALRI were included (mean age: 9.5 months; IQR: 4–22 months); 96.6% were tested for viral detection, and 66.4% were positive (3877 cases). Respiratory syncytial virus (RSV) was the most prevalent. Influenza showed typical winter seasonality in 2018, 2019, and 2023. However, 2022 exhibited a bimodal pattern: late summer and spring, with co‐circulation of influenza A and B in the second peak. CFR varied by viral diagnosis; influenza showed the highest CFR, all deaths related to influenza A. Among 354 influenza cases, 81% were < 5 years old, 53% were male, 63% had comorbidities, and 14.1% required intensive care. Mean of influenza vaccine coverage (6–24 months) was 21.4%. In both periods, patients with influenza were more likely to have pneumonia. Additionally, in the postpandemic period, malnourishment or being 3 years of age or older was also associated with a higher likelihood of influenza infection compared with infection with other respiratory viruses.

**Conclusions:**

Influenza primarily affected children under 5 years old. Postpandemic cases involved older individuals, and increased circulation of influenza A H3N2 was observed. Vaccination coverage was notably low. Influenza returned to its usual seasonal pattern in 2023.

## 1 Introduction

Influenza illness in humans is primarily caused by types A and B, both of which are responsible for seasonal epidemics and can lead to severe respiratory symptoms. Each year, mainly during the winter season, influenza affects 1 billion people worldwide. The World Health Organization estimates that each year there are between 3 and 5 million cases of severe illnesses and between 290,000 and 650,000 respiratory deaths [1]. Both types of influenza can cause significant morbidity and mortality, particularly during seasonal flu epidemics, which affect vulnerable populations such as young children, the elderly, and individuals with underlying health conditions [2]. Globally, in recent years, the most prevalent circulating subtypes of influenza A have been A(H1N1)pdm09 and A(H3), whereas influenza B has predominantly been of the Victoria lineage [3].

Acute lower respiratory tract infections (ALRIs) not only result in a high mortality burden but also have a significant impact on medication use, absenteeism, and healthcare service utilization [4, 5]. ALRIs were the second leading cause of death in children under 5 globally from 2000 to 2021, causing around 784,600 deaths in 2021 [6]. Influenza accounts for 7% of ALRI cases, 5% of ALRI hospitalizations, and 4% of ALRI deaths in children under 5, with 82% of mortality occurring in developing countries [7]. In Argentina, respiratory illness was the fourth leading cause of death in children under 5, representing 3.5% of total deaths in this age group with a rate of 4.7 per 100,000 [8].

A worse outcome of H1N1 influenza infection has also been described in pediatrics, with a higher frequency of cough and seizures compared with H1N1 infection in the pre‐COVID‐19 period [9, 10]. Since early 2020, changes in contact and mobility patterns due to COVID‐19 isolation have disrupted the typical seasonal cycles of many infectious diseases worldwide, including influenza. Influenza began to resurge toward the end of 2021, with activity occurring outside the usual season in the Southern Hemisphere [11].

Influenza vaccination is the most effective method to prevent influenza infection and its complications. Vaccine effectiveness varies each season depending on circulating influenza strains and vaccination rates [12]. Between 2011–2020, the US Influenza Vaccine Effectiveness Network demonstrated a pooled VE for any influenza of 46% (95% CI: 43–50), with an overall and by type/subtype, VE against influenza illness highest among children in the 6‐ to 59‐month age group compared with older pediatric age groups. VE was lowest for influenza A(H3N2) virus infection [13]. In Argentina, the influenza vaccine has been mandatory and free for all children aged 6 to 24 months since 2011, administered in a two‐dose schedule with a 1‐month interval.

Given the impact of ALRIs in pediatrics, it is important to analyze the risk factors associated with complications and mortality from this disease. Identifying these factors will allow for the adoption of measures to control or reduce them, as well as identifying patients at risk of severe ALRIs, thus providing appropriate care.

This multicenter study aims to describe the clinical and epidemiological profile of pediatric patients hospitalized for ALRIs of any viral cause and for influenza before (2018–2019) and after (2022–2023) the COVID‐19 pandemic.

## 2 Materials and Methods

### 2.1 Cross‐Sectional Multicenter Study

All patients hospitalized for ALRIs under the age of 18, detected through the Epidemiological Surveillance Program (PVE) year round, were included in five tertiary centers in Argentina in four seasons 2018, 2019, 2022, and 2023. The participating centers are in three different regions of the country: Central region: R. Gutiérrez Children's Hospital (Buenos Aires city), “Profesor Alejandro Posadas” National Hospital (Buenos Aires Province), and Dr. Víctor J. Vilela Children's Hospital (Rosario, Santa Fe); Andes Region: Notti Hospital (Mendoza); and Northeast region: “Fernando Barreyro” Pediatric Hospital (Posadas, Misiones).

Epidemiological Surveillance Program (PVE) conducts active surveillance of patients hospitalized for ALRI with testing for viral etiological diagnosis in all cases as routine practice in each hospital.

Demographic data (age, sex, residence), clinical information (clinical presentation) (bronchiolitis, pneumonia), comorbidities (chronic or recurrent respiratory disease, malnutrition, congenital heart disease, genetic condition, neurological disease, and immunosuppression), clinical course (discharge, transfer to a different facility, death), treatment, duration of hospitalization, and history of influenza vaccination through hospital records or patient vaccination cards were recorded.

The presence of any of the following conditions was recorded as chronic or recurrent respiratory disease: recurrent obstructive bronchitis or asthma, gastroesophageal reflux, cystic fibrosis, bronchopulmonary dysplasia, recurrent pneumonia, and laryngitis.

Acute lower respiratory tract infection (ALRI) included bronchiolitis (first episode of wheezing associated with clinical evidence of viral infection in a child under 2 years old) or pneumonia (acute infection of the lung parenchyma with clinical signs of alveolar occupation).

### 2.2 Etiological Diagnosis

All patients hospitalized for ALRIs were tested for respiratory viruses. During the seasons 2018 and 2019, respiratory virus rapid diagnostic tests were mainly used, employing indirect immunofluorescence assay (IFA) technique (Light Diagnostics Respiratory Panel I. Screening and viral identification IFA, Chemicon [Millipore]) on nasopharyngeal secretions obtained by nasogastric tube suction for detection of the following respiratory viruses: adenovirus, respiratory syncytial virus, influenza A and B, parainfluenza 1, 2, and 3. Genetic characterization of influenza A virus detected by IFA was performed by RT‐PCR according to WHO‐recommended protocols [14]. Since 2022, RT‐PCR technique has been used for virological diagnosis in all centers.

### 2.3 Data Analysis

All hospitalized ALRI cases were included. A general description was conducted to characterize ALRI and influenza cases. Epi Info version 7 (US Centers for Disease Control and Prevention, Atlanta, GA, USA) was used for data analysis. Categorical variables were analyzed using the chi‐square test with Yates correction. The measure of association used was odds ratio (OR) with a 95% confidence interval (CI). Bivariate analysis was conducted to compare the clinical‐epidemiological characteristics of influenza cases before and after the SARS‐CoV‐2 pandemic. Risk factors associated with influenza infection compared with other viruses were then analyzed, followed by multivariate analysis to establish independent predictors using logistic regression model of SPSS software version 15.0 (SPSS Inc., Chicago, IL, USA). A probability less than 0.05 was considered significant. Comparative analysis between influenza A and B was also performed.

### 2.4 Ethical Considerations

The privacy rights of patients were observed in all cases, in accordance with the World Medical Association's Declaration of Helsinki, the International Code of Ethics for experiments involving humans. The study was approved by the Ethics and Research Committees of each participating center. This study will not affect human rights or cause harm to the environment, animals, or future generations. All files were anonymized prior to analysis, and a restricted access database was created for researchers at each center.

## 3 Results

During the study period, a total of 5838 cases of ALRIs were included, with 41.3% (2412) recorded during the prepandemic period (2018–2019) and 59.7% (3426) after it (2022–2023). In the postpandemic period, there was a higher positivity in viral detection (pre: 49.6%; post: 78.2%; *p* < 0.001). The most prevalent virus throughout the study period was RSV followed by parainfluenza and influenza. In the postpandemic period, there was a significant decrease in the prevalence of RSV (67.3% vs. 44.1%; *p* < 0.001) and an increase in parainfluenza (6.9% vs. 12.0%; *p* < 0.001) and metapneumovirus (5.9% vs. 12.1%; *p* < 0.001) (Table 1).

**TABLE 1:** ALRI annual cases distribution by viral detection and seasonality.

Total ALRI (*n* = 5838)	Pre‐pandemic		Post‐pandemic	
	2018 (*n* = 1220)	2019 (*n* = 1192)	2022 (*n* = 1690)	2023 (*n* = 1736)
**Viral detection**				
Positivity rate	623 (51.1%)	574 (48.1%)	1303 (77.1%)	1377 (79.3%)
RSV	71.7%	62.5%	35.5%	52.3%
Influenza A	3.7%	10.5%	6.6%	7.6%
Influenza B	2.7%	0.5%	4.8%	0.0%
Parainfluenza	5.8%	8.0%	10.8%	13.1%
Adenovirus	3.0%	6.6%	6.8%	6.1%
Metapneumovirus	6.6%	5.2%	17.3%	12.1%
**Seasonality (EW median; IQR)**				
ALRI all causes	29 (26–34)	26 (20–31)	28 (22–38)	24 (19–34)
ALRI influenza positive	34 (32–38)	26 (24–27)	41 (17–44)	26 (23–25)

Abbreviations: EW: epidemiological weeks; IQR: interquartile range.

**FIGURE 1:**
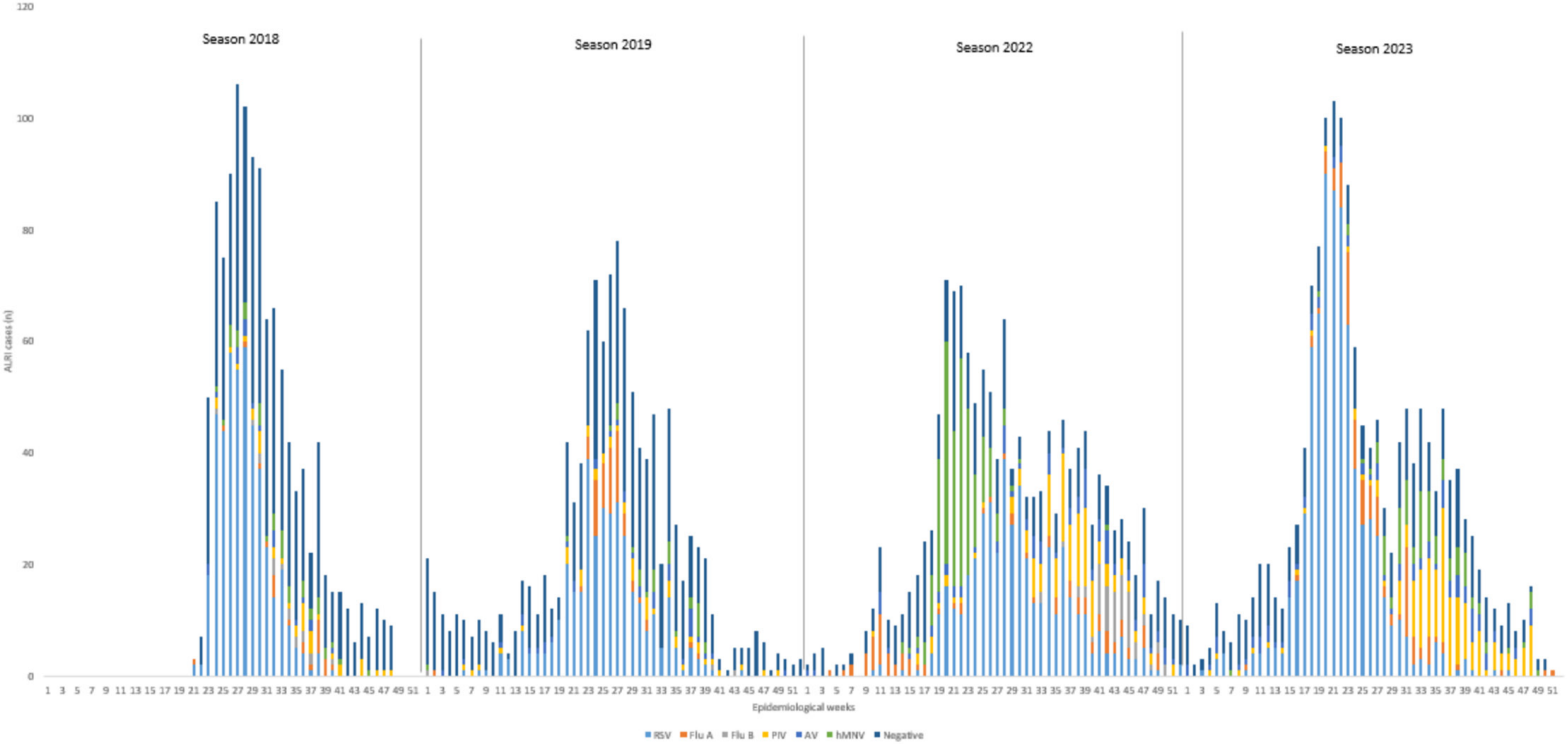
Epidemic curve of hospitalized cases due to acute lower respiratory tract infection (ALRI) caused by RSV, influenza, parainfluenza, adenovirus, and human metapneumovirus. Years 2018–2023. References: RSV: respiratory syncytial virus; Flu A: influenza A; Flu B: influenza B; PIV: parainfluenza virus; ADV: adenovirus; hMNV: human metapneumovirus.

ALRI seasonality showed differences between both periods, with a shift of 6 weeks forward in the postpandemic period compared with the prepandemic period (Figure 1). During the year 2022, there was a winter peak of ALRIs characterized by high positivity of metapneumovirus, which was the most prevalent virus during the early weeks of that season, displacing RSV. In 2023, RSV regained the top position and its usual seasonality with an exponential increase in the early weeks of circulation.

**FIGURE 2:**
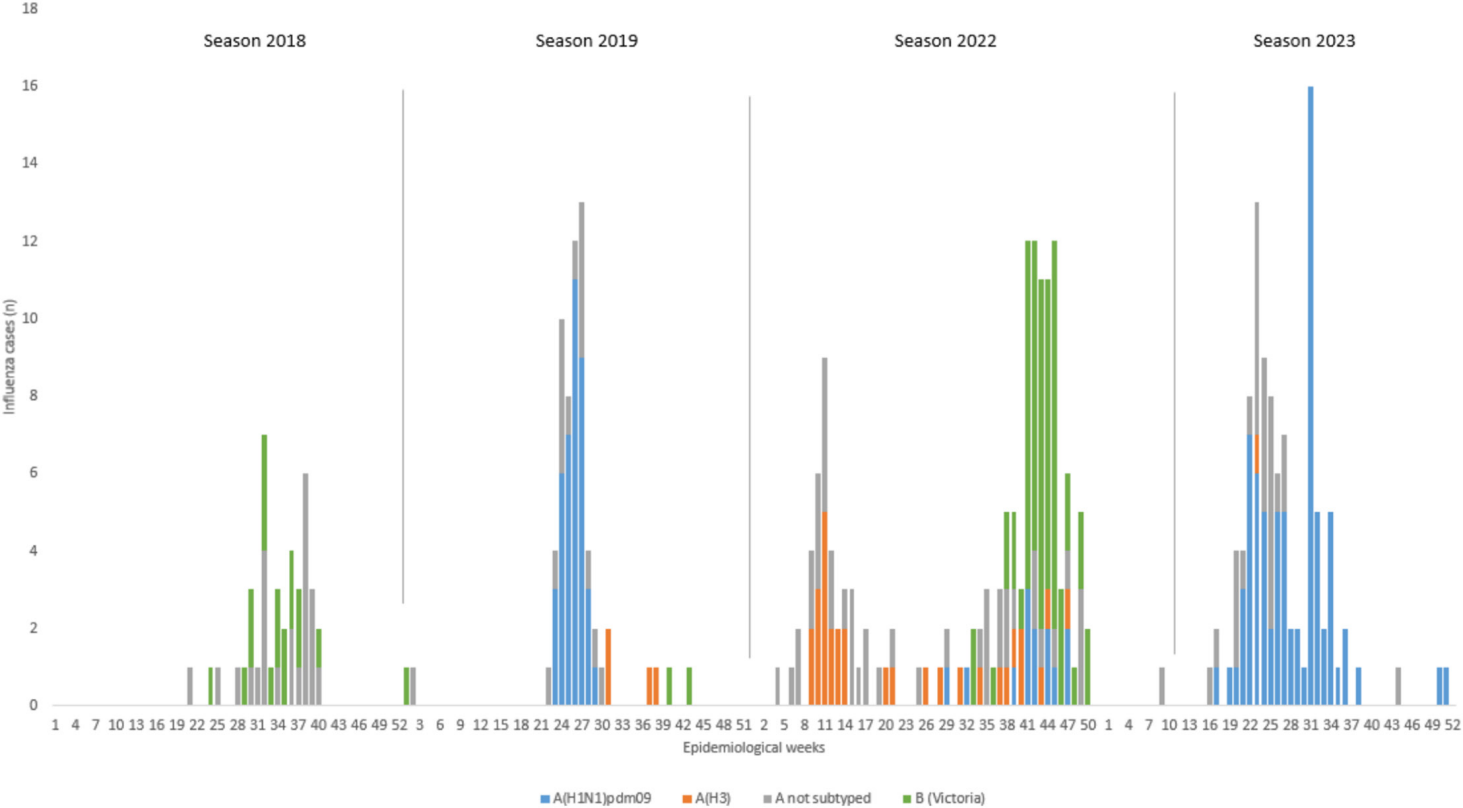
Epidemic curve of hospitalized cases with acute lower respiratory tract infection (ALRI) positive for influenza by types and subtypes per epidemiological week. Years 2018–2023. Note: “not subtyped” refers to samples with a Ct value too high for subtype determination, indicating that the viral load was insufficient for successful subtyping rather than the absence of a subtype.

In relation to influenza, subtypes A and B also exhibited differential circulation patterns. While maintaining a winter seasonality pattern the subtypes circulated differentially, accentuated in the postpandemic period during which they circulated with an atypical (bimodal) seasonality. Influenza B circulates later in the winter months, after influenza A (Figure 2).

The most prevalent type during the 4 years evaluated was influenza A, accounting for 70%. However, the distribution was not homogeneous considering that type B (Victoria) was recorded in 42.5% of cases in 2018 and 41.8% in 2022, whereas no cases of influenza B were recorded in 2023, and it represented only 4.2% in 2019.

Regarding influenza A subtypes, the predominant one was H1N1(pdm09), representing 36.1%, also with differences between the different years (Table 2). Viral coinfection was recorded in 25.3% of cases, with the most frequent coinfection being with RSV (46%), followed by rhinovirus (20.7%), parainfluenza (16.1%), metapneumovirus (9.2%), and adenovirus (8%).

**TABLE 2:** Distribution of ALRIs by influenza type and subtype per year. 2018–2023.

Influenza types and subtypes	2018		2019		2022		2023		2018–2023	
	*n*	%	*n*	%	*n*	%	*n*	%	*n*	%
Flu A not subtyped^a^	23	57.5	16	25.4	43	29.1	28	27.2	110	31.1
Flu A H3N2	0	0	4	6.3	30	20.3	0	0	34	9.6
Flu A H1N1(pdm09)	0	0	40	63.5	13	8.8	75	72.8	128	36.2
Flu B (Victoria)	17	42.5	3	4.8	62	41.9	0	0	82	23.2
Influenza (A and B)	40	100	63	100	148	100	103	100	354	100

^a^ “Not subtyped” refers to samples with a Ct value too high for subtype determination, indicating that the viral load was insufficient for successful subtyping rather than the absence of a subtype.

The median age of influenza cases varied across the different years analyzed. Between 70%–80% of cases occurred before the age of 5 years. Pneumonia was the most frequent clinical presentation in hospitalized cases, and the most common signs and symptoms were cough and fever, followed by respiratory difficulty. Approximately 15% required intensive care, 90% of cases required supplementary oxygen treatment, 38% received empirical antibiotic treatment upon admission, and 16.5% discontinued antibiotic treatment upon positive virological result. Mean of influenza vaccine coverage (6–24 months) was 21.4% (15.6–31.2) for complete schedule according age (Table 3).

**TABLE 3:** Clinical and epidemiological characteristics of influenza cases.

Influenza (*n* = 354)	Pre‐pandemic (2018 and 2019)		Post‐pandemic (2022 and 2023)	
	2018 (*n* = 40)	2019 (*n* = 63)	2022 (*n* = 147)	2023 (*n* = 104)
Age in months (median; IQR)	16.5 (10–37)	8 (5–12)	25 (8–70)	12.7 (5.6–41)
Sex (male)	21 (52.5%)	34 (54%)	76 (51.7%)	57 (54.8%)
Age groups				
< 6 months	3 (7.5%)	19 (30.2%)	25 (17.1%)	29 (27.9%)
6–11 months	10 (25%)	27 (42.9%)	21 (14.4%)	21 (20.2%)
12–23 months	12 (30%)	12 (19.0%)	24 (16.4%)	19 (18.3%)
2–4 years	9 (22.5%)	3 (4.8%)	33 (22.6%)	19 (18.3%)
5–9 years	6 (15%)	1 (1.6%)	30 (20.5%)	11 (10.6%)
10 years or more	0 (0%)	1 (1.6%)	13 (8.9%)	5 (4.8%)
Influenza vaccine coverage				
6–24 months of age	5 (31.2%)	6 (15.4%)	11 (28.2%)	5 (15.6%)
Clinical features				
Pneumonia	29 (72.5%)	38 (60.3%)	89 (60.5%)	67 (64.4%)
Cough	28 (70%)	50 (79.4%)	98 (66.7%)	71 (68.3%)
Fever	37 (92.5%)	50 (79.4%)	132 (89.8%)	72 (69.2%)
Respiratory distress	31 (77.5%)	59 (93.6%)	45 (30.6%)	74 (71.1%)
Vomiting	4 (10%)	1 (1.6%)	20 (13.6%)	7 (6.7%)
Rhinorrhea	19 (45.5%)	46 (73%)	74 (50.3%)	56 (53.8%)
Treatment and evolution				
Received empirical antibiotic treatment upon admission	39 (97.5%)	55 (87.3%)	26 (17.8%)	15 (14.6%)
Empirical antibiotic treatment was suspended with a positive viral diagnosis	10 (35.7%)	8 (27.6%)	11 (8.7%)	18 (19%)
Supplemental oxygen requiring	37 (92.5%)	62 (98.4%)	121 (83.4%)	97 (93.3%)
Length of hospitalization in days (mean; IQR)	8 (5–11)	9 (6–14)	7 (5–10)	7 (5–11)
Intensive care	9 (22.5%)	11 (17.5%)	15 (10.5%)	15 (14.7%)
Case fatality rate	0 (0%)	1 (1.6%)	3 (2.1%)	1 (1%)

The case fatality rate (CFR) varied depending on the viral detection: There were 30 deaths out of 5734 cases overall, 5 deaths out of 269 cases due to influenza A, and 3 deaths out of 1950 cases due to RSV. No deaths related to influenza B were reported. Influenza A CFR varied in different years (2018: 0%; 2019: 1.7%; 2022: 3.5%; 2023: 1%). Five children between 6 months and 11 years old died (1 with H3N2 and 4 not typed), four of them with comorbidities (chronic neurological disease, asthma, or immunosuppression).

Univariate analysis of the characteristics of ALRI cases due to influenza in the prevaccination and postvaccination periods are shown in Table 4.

**TABLE 4:** Clinical and epidemiological characteristics of influenza cases comparing pre (2018–2019) and post‐pandemic (2022–2023) periods.

Influenza cases features	Influenza		
	Pre‐pandemic	Post‐pandemic	*p*
	2018–2019	2022–2023	
	*N* = 103	*N* = 250	
	% (*n*)	% (*n*)	
Age in months (median; interquartile range)	10 (6–19 months)	20 (7–57 months)	1.000
Influenza A H1N1	38.8% (40)	34.9% (88)	0.486
Influenza A H3N2	3.9% (4)	12.3% (31)	0.015
Influenza A not subtyped	37.9% (39)	28.2% (71)	0.073
Influenza B (Victoria)	19.4% (20)	24.6% (62)	0.293
Epidemiological week of the start of influenza circulation onset	27	31	0.190
Complete vaccination for age	18.5% (18)	32.9% (61)	0.010
Comorbidities	65% (67)	62% (156)	0.608
Pneumonia	50.5% (52)	70.9% (178)	< 0.001
Received empirical antibiotic treatment upon admission	91.2% (94)	16.5% (41)	< 0.001
Empirical antibiotic treatment was suspended with a positive viral diagnosis	19.1% (18)	70.3% (29)	< 0.001
Intensive care requirement	19.4% (20)	12.2% (30)	0.081
Case fatality rate (CFR)	0.9%	1.6%	0.638

Table 5 shows the multivariate analysis of factors associated with ALRI caused by influenza compared with other respiratory viruses in the prepandemic and postpandemic periods. In both periods, patients with influenza were more likely to have pneumonia. Additionally, in the postpandemic period, being 3 years of age or older was also associated with a higher likelihood of influenza infection compared with infection with other respiratory viruses.

**TABLE 5:** Multivariate analysis of factors associated with influenza infection compared with other respiratory viruses in hospitalized ALRI cases with viral diagnosis before and after the pandemic.

Factors associated with ALRI caused by influenza	OR	95% CI	*p*
**Prepandemic period**			
**Pneumonia as clinical presentation**	**2.05**	**1.33–3.16**	**< 0.001**
Malnourishment	1.83	0.94–3.56	0.075
3 years old or older	0.58	0.30–1.09	0.090
Congenital heart disease	0.90	0.45–1.81	0.785
Chronic neurological disease	1.90	0.91–3.96	0.084
Chronic or recurrent respiratory disease	1.15	0.77–1.72	0.473
Sex (male)	0.87	0.54–1.41	0.591
History of previous hospitalization	1.21	0.76–1.93	0.411
**Postpandemic period**			
**Pneumonia as clinical presentation**	**2.15**	**1.56–2.95**	**< 0.001**
**Malnourishment**	**1.84**	**1.01–3.38**	**0.046**
**3 years old or older**	**1.75**	**1.28–2.38**	**< 0.001**
Congenital heart disease	0.91	0.55–1.49	0.709
Chronic neurological disease	0.80	0.44–1.43	0.456
Chronic or recurrent respiratory disease	0.77	0.59–1.00	0.059
Sex (male)	0.75	0.55–1.02	0.068
History of previous hospitalization	0.92	0.68–1.24	0.596

*Note*: Variables in bold are statistically significant.

## 4 Discussion

In our study, it is observed that in Argentina, as in other parts of the world, the COVID‐19 pandemic altered the patterns of viral circulation, including the influenza virus [1, 3]. The historical curve of positive cases of respiratory viruses showed a marked decline for the year 2020, registering the presence of adenovirus and isolated cases of RSV in 2021 and a return of circulation in 2022, with unusual behavior in both seasonality and the number of recorded influenza cases [15, 16].

The widespread use of nonpharmaceutical interventions (NPIs), during the COVID‐19 pandemic, which helped reduce the circulation of SARS‐CoV‐2, also impacted the transmission of other respiratory pathogens [17], potentially creating an “immunological debt” [18]. This concept suggests that the reduced exposure to various pathogens during the pandemic may have affected the immune system's “trained immunity,” which normally strengthens responses to subsequent infections. This “debt,” along with the lower vaccination coverage recorded during the NPI periods, could have contributed to the resurgence of respiratory viruses, such as metapneumovirus and influenza B, in 2022, and could increase the likelihood of future epidemics [19].

In the epidemic curve of the analyzed cases, we observe in the postpandemic period the appearance of metapneumovirus displacing RSV in 2022 and an early seasonal activity of the latter in 2023. There is also a notable increase in the prevalence of parainfluenza circulation, as observed in other parts of the world [1, 3].

Regarding influenza, it only regained its usual winter seasonality characteristic of temperate climates in 2023. It is noteworthy that in 2022, an atypical bimodal curve with co‐circulation of influenza A and B was observed [20]. This “influenza rebound” was seen in both the northern and southern hemispheres. Specifically, in South America in 2022, seasonality was atypical with early activity, unlike the delayed activity in the north [21]. As in Australia, in our study, influenza appeared in the summer after the exponential peak of Omicron (B.1.1.529), with reports of co‐circulation of SARS‐CoV‐2 and influenza A [22].

During the postpandemic period, we observed greater circulation of influenza A H3N2, similar to what was described by the CDC, which reported that in the 2022–2023 season, 95.4% of infections were with influenza A virus; 80.2% of those subtyped were A(H3N2) and 19.6% were A(H1N1)pdm09 [23]. This perhaps shows that the synchrony in influenza circulation between hemispheres has been restored. The restoration could be due to factors such as increased global mobility, the easing of COVID‐19 restrictions, similar environmental conditions, viral evolution, and the reintroduction of circulating strains.

Seasonality, which is a characteristic of influenza virus circulation, according to Temermious et al., is a complex phenomenon constructed by the interaction between people's contact patterns, the virus's survival characteristics, and host immunity [24]. We must also consider the role that climate change might play in this phenomenon [25]. In alignment with other regions characterized by a temperate pattern of influenza circulation, influenza A epidemics were observed during both summer and winter, whereas influenza B epidemics occurred primarily in winter and spring [26].

In this analysis of seasonal dynamics, we cannot overlook that one of the limitations of our work is the use of nonmolecular detection techniques in the prepandemic period. Although this is a limitation, it allows us to evidence the positive impact that the COVID‐19 pandemic had on the epidemiological surveillance of respiratory viruses in the real world, improving access to detection through molecular tests [27]. The sensitivity of indirect IFA for detecting viruses in respiratory samples varies depending on the type of virus. For example, one study found that IFA has good sensitivity for RSV or influenza but fails to detect other respiratory viruses [28, 29]. This marks the need to redouble efforts to maintain these diagnostic resources over time. Better access to viral diagnosis could also have impacted the reduced use of antibiotics at patient admission for ALRI observed in the postpandemic period or the greater discontinuation of antibiotics when a positive influenza diagnosis was available. This could also positively affect the rational use of antimicrobials, helping to reduce multidrug resistance [30, 31, 32].

During the years analyzed, the highest prevalence of influenza was observed in children under 5 years old, a group primarily involved in disease transmission [33]. Specifically, age over 3 years was an independent predictor of influenza detection versus other viruses in hospitalized patients with ALRI.

Clinical adverse outcomes are generally worst for youngest children (< 5 years) and in adults at older ages [34]. In our study, we observed a high prevalence of the disease as pneumonia, especially in the postpandemic period (71%), and higher CFR in the postpandemic period, although with non‐significant differences in this last indicator. The American Academy of Pediatrics noted that in the United States, half of the pediatric deaths from influenza were observed in children without comorbidities, and 90% were not fully vaccinated. Approximately 15% of our patients required critical care, similar to reports from 14 US Influenza Hospitalization Surveillance Network sites [35].

This opens the debate on the target population for influenza vaccination in different regions of the world and the impact on vaccination coverage post‐pandemic, considering that the costs‐of‐illness are generally higher in UMICs than in LMICs/LICs, but the highest national economic burden, as a percent of gross domestic product and national health expenditure, was reported from an LIC [36]. Vaccination is one of the most effective measures to prevent influenza illness and its complications [37]. The World Health Organization estimated that since 1974, vaccination has averted 154 million deaths, including 146 million among children younger than 5 years of whom 101 million were infants younger than 1 year [38]. Compared with unvaccinated individuals, lower odds of ICU admission were found for partially vaccinated children (aOR 0.64 [95% CI 0.44–0.92]) and fully vaccinated children (0.52 [0.28–0.98]) [39].

This indicates that equitable access to vaccination remains crucial to sustain health gains. In the Americas region, 39 (89%) of the 44 countries/territories reporting data to the Pan‐American Health Organization have policies for seasonal influenza vaccination; 77% of the countries have vaccination for children, with differences in the age groups included: 19% vaccinate between 6 months‐2 years (including Argentina), 19% between 6 months‐3 years, 32% between 6 months‐5 years, and 26% in other age groups [40]. Nogareda et al. also highlight the drop in influenza coverage of 9% in children in the Americas region [40]. In our study, suboptimal coverage was observed in both periods, similar to the country's report of 42% coverage in 2021 compared with 75% in 2019. Sustaining influenza vaccination programs is critical for the control of future influenza seasons. As mentioned in the Global Influenza Strategy 2019–2030 by the World Health Organization, “The threat of pandemic influenza is ever‐present. The ongoing risk of a new influenza virus transmitting from animals to humans and potentially causing a pandemic is real. The question is not if we will have another pandemic, but when” [41].

Epidemiological surveillance of acute lower respiratory infections is essential for preparation, detection, and response. Sentinel surveillance in tertiary referral hospitals in the country shows an adequate correlation with what is observed locally and internationally.

## Author Contributions

**Angela Gentile:** conceptualization, methodology, writing – original draft, writing – review and editing, project administration, supervision, resources. **María del Valle Juárez:** conceptualization, methodology, investigation, validation, visualization, writing – original draft, writing – review and editing, software, formal analysis, data curation, supervision. **Gabriela Ensinck:** writing – review and editing, investigation, supervision. **Oscar Lopez:** investigation, writing – review and editing, supervision. **Pablo Melonari:** investigation, writing – review and editing, supervision. **Tatiana Fernández:** investigation, data curation. **Andrés Gioiosa:** investigation, data curation. **Gustavo Lazarte:** investigation, data curation. **Silvina Lobertti:** investigation, data curation. **María Florencia Lucion:** conceptualization, writing – review and editing, formal analysis. **Natalia Pejito:** investigation, data curation. **Camila Racana:** investigation, data curation. **Leandro López:** investigation, data curation. **Gabriela Gregorio:** writing – review and editing, investigation, supervision.

## Conflicts of Interest

The authors declare no conflicts of interest.

### Peer Review

The peer review history for this article is available at https://www.webofscience.com/api/gateway/wos/peer‐review/10.1111/irv.70078.

## Data Availability

The data that support the findings of this study are available from the authors upon request.
